# A combined methodological approach to characterize pig farming and its influence on the occurrence of interactions between wild boars and domestic pigs in Corsican micro-regions

**DOI:** 10.3389/fvets.2024.1253060

**Published:** 2024-04-02

**Authors:** Liane Dupon, Bastien Trabucco, Facundo Muñoz, François Casabianca, François Charrier, Morgane Laval, Ferran Jori

**Affiliations:** ^1^Laboratoire de Recherche sur le Développement de l’Elevage, INRAE, Corte, France; ^2^UMR SELMET, CIRAD-INRAE, Montpellier, France; ^3^CIRAD, UMR ASTRE, Montpellier, France; ^4^ASTRE, Univ Montpellier, CIRAD – INRAE, Montpellier, France; ^5^UMR LISIS, INRAE – Université Gustave Eiffel, Marne-la-Vallée, France

**Keywords:** biosecurity, incursion, outdoor farming, pig production, *Sus scrofa*, typology, wildlife

## Abstract

The pig sector in Corsica is based by a wide range of farming systems, mainly characterized on traditional extensive practices, which favor contacts between domestic and wild individuals. These contacts are suspected to influence the maintenance and the transmission of shared infectious diseases between both populations. Therefore, it is important to develop methods that allow to understand and anticipate their occurrence. Modeling these interactions requires accurate data on the presence, location and use of land on pig farms and farming practices, but such data are often unavailable, incomplete or outdated. In this study, we suggest a method to collect and analyze pig farming information that combines approaches from social sciences and epidemiology and enables a spatial representation of an index of potential interaction (IPI) between wild and domestic pigs at municipality level in the Corsican territory. As a first step of the process, interviews were conducted to gather information from 103 pig farms. Then, using hierarchical clustering, we identified five different clusters of pig farming practices which were evaluated and validated by local experts using participatory tools. The five pig farming clusters with their respective estimated levels of direct and indirect interactions with wild boars were combined in a linear equation with pig density to estimate a hypothetical index of potential interaction (IPI) in 155 municipalities. Our results revealed the diversity of pig farming practices across the island of Corsica and pointed out potential hotspots of interaction. Our method proved to be an effective way to collect and update information on the presence and typology of pig farms which has the potential to update official livestock production statistics. The spatial representation of an IPI between wild boars and domestic pigs in the Corsican territory could help design regional disease management strategies and policies to improve the control of certain shared pig pathogens in pig farms from Corsica.

## 1 Introduction

Recent episodes of emergence, re-emergence or persistence of animal infectious diseases has drawn scientific attention to the wildlife-livestock interfaces as a key factor to improve our understanding of shared pathogen dynamics (1–3). The interest on interactions between wild boars and domestic pigs has particularly grown with the global spread of African swine fever across the world (4–7). However, other diseases shared between wild boars and domestic pigs jeopardize disease eradication efforts in the pig sector, while affecting the health of wild boar populations of and representing a potential public health risk (8–13). Moreover, the recent increase of consumer demand for outdoor farming products in developed economies has raised concerns about the biosecurity of open production systems in general, and about potential interactions between domestic and wild/feral pigs (2, 9, 14–17). Understanding the different drivers of interactions between populations of wild and domestic pigs requires analysis of the infectious interface using approaches from different disciplines (3). Such approaches often include ecological, epidemiological or sociological methodologies focused on a farm perimeter, water points, or the edge of a protected area, whereas fewer studies have addressed the risk of wildlife-livestock interactions and pathogen spill-over at a larger geographical scale (18–21).

Because of their ancestral tradition of outdoor pig farming, Mediterranean habitats are particularly prone to interactions between domestic pigs, feral swine and wild boars. For instance, free ranging farming systems in Sardinia have been held accountable for the persistence of African swine fever for decades (5, 22, 23), while in the Iberian Peninsula, the co-existence of Iberian pigs with a large wild boar population in extensive estates is considered as a risk for the re-emergence of Aujeszky’s disease or the maintenance of bovine tuberculosis (18, 20, 24). The French Island, Corsica, is an example of specific socio-ecological context favoring different types of direct and indirect sexual, trophic and agonistic interactions between wild and domestic pigs and the resulting dissemination of shared porcine pathogens among these populations (16, 25). These include endemic diseases and re-emerging or recent diseases that can have a serious impact on livestock productivity and public health such as classical swine fever (26), Aujeszky’s disease (15), trichinellosis (27), toxoplasmosis (28) or hepatitis E virus (8).

Several authors have characterized the type, frequency, intensity and location of interactions between wild boar and domestic pigs, which are significantly influenced by hunting and farming practices (8, 15). However, the whole range of outdoor farming systems and the potential impact of their spatial distribution on the probability of interaction with wild boars has not been well characterized in Corsica to date. Given the variety of landscapes and the distribution of resources in Corsica, we hypothesize that some regions of the island with specific ecological features or forms of land use are prone to certain types of pig management practices that facilitate these interactions. Nevertheless, studying such complex interface at a territorial scale is challenging as data on farming practices and specific locations of farming systems is often inaccurate. A possible approach to address this challenge is to rely on local knowledge and expertise (29, 30), with the implementation participatory epidemiology methods (31, 32). As several epidemiological and zootechnical information was already available in Corsica from previous studies (15–17, 25), we decided to combine different geographical, epidemiological and zootechnical approaches to conduct a spatial analysis of farming systems that could be used as an indicator of spatial potential interaction patterns.

The specific purpose of our work was to explore new methodologies, combining participatory approaches and analysis of zootechnical data, to represent the distribution of pig management practices at the scale of some Corsican micro-regions and their potential risk of interactions with wild boar based on pig farming.

## 2 Materials and methods

### 2.1 Study area and context

The island of Corsica is located in the Mediterranean Sea off the coast of the South of France and covers 8,722 square kilometers. Its altitude (ranging from 0 to 2,706 m and 568 m on average) and landscape characteristics are emblematic of Corsican identity (33). The variability of soils and topography in the Island enables the adoption of a diversity of crop and livestock production systems (34). In 2015, the French Ministry of Agriculture and Food defined 16 micro-regions resulting from the aggregation of the 30 small natural regions originally defined in the 1979 agricultural census (35) and based on homogeneity and natural limits criteria (see Aggregated Small Natural Regions, Supplementary material S1). Based on these criteria, we defined the term micro-region as a division of the territory based on certain homogenous geographical characteristics that influenced its land use and agricultural production practices and used this classification throughout this article.

In the past, pig farming was widespread in Corsica. The traditional Corsican pig farming system, which consisted fundamentally on free-ranging systems of backyard animals for family consumption, is based on the exploitation of sylvo-pastoral resources by a local breed of slow growing pigs. Pigs aged 18–24 months are slaughtered in winter after a period of free ranging in autumn and winter to finish their fattening with acorns and chestnuts (36). In some areas, farmers also keep their pigs in mountain pastures in summer (17, 25, 37). The Corsican pig sector consequently has a strong link with certain micro-regions featuring particular ecological landscapes such as mountain pastures or chestnut forests. Today, especially thanks to PDO (Protected Designation of Origin) certification and use of the “Nustrale” breed, the production of Corsican dry cured meat (*charcuterie*) is prized for both its quality and flavor.

In Corsica, domestic and wild swine populations are largely represented in terms of their distribution and suspected abundance (38, 39) encompassing an interesting genetic diversity composed of different domestic pigs’ breeds, feral pigs, wild boars and cross-bred individuals. Although the proportion of cross-bred animals in this population has not been accurately quantified, it was estimated to reach 55% in some regions during the 1980’s (11, 40).

### 2.2 Study design

**Figure 1:**
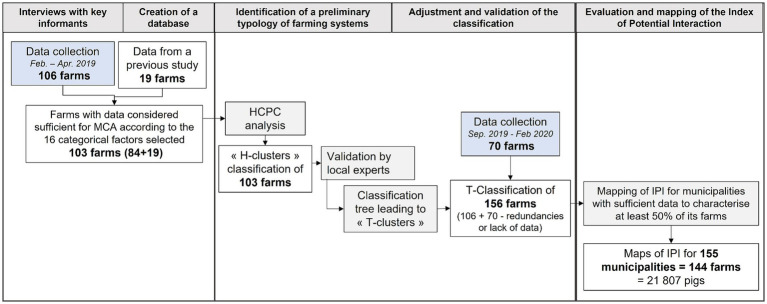
Summary of the methodology used and the number of farms covered.

Given the diversity and heterogeneity of farming practices, we hypothesized that the potential contribution of pig farming to the probability of occurrence of interactions with wild boar was multi-factorial (41). Based on previous work in Corsica (16) and other pig farming locations (42–44), we first identified key zootechnical practices involved in the occurrence of different types of interaction and defined a method based on the clustering of farming practices. The main steps to comply with this process were the following: (i) Implementation of interviews with key informants in order to collect regional data on formal and informal pig production; (ii) Creation of a reliable database combining information collected through interviews with existing data and technical knowledge; (iii) Hierarchical clustering on principal components (HCPC) based on multiple correspondence analysis (MCA) to identify a preliminary typology of farming systems (clusters) based on the use of distinct pig farming practices; (iv) Adjustment of our preliminary farm typology by a group of local experts (38) to determine the main factors that describe the clusters and determination of the IPI associated to each cluster; (v) Validation of a new classification of the clusters undertaken with local stakeholders and a classification tree method; (vi) Evaluation and mapping of IPI related to pig farming at the municipal scale, using pig density and the IPI in each cluster. A summary of the methodology is shown in Figure 1.

#### 2.2.1 Data collection

Data collection was organized on semi-structured interviews with key informants (30, 45) in two different periods: the first from February to April 2019 and the second from September 2019 to February 2020. The questionnaire was designed to gather regional information on formal and informal data on pig production farms from local key informants, including pig density, land area occupied by pigs, and the main pig farming practices. All the semi-structured interviews were conducted by the same interviewer at the place where the key informants lived or worked. Key informants were selected on the basis of their experience in livestock farming, their involvement in local farming organizations, and on the recommendation of other informants or stakeholders in pig farming. The interviews allowed to compile a final list of 176 farms (106 farms during the first period and 70 farms during the second). After the first period, only 103 of those farms had sufficient quantity and quality of data to perform the HCPC analysis, including 84 farms from the first data collection period and 19 farms from data available from a previous study (16) were retained (Figure 1).

#### 2.2.2 Selection of farming practices

We focused our selection of factors on free-range pig farming, the permeability of fences and management of feed and waste taking into account direct and indirect wild-domestic pig interactions (25). Because direct interactions are often driven by sexual and agonistic behavior, we focused on farming practices linked with reproductive management, such as castration or spaying of pigs not intended for breeding.

For the MCA analysis, we selected 16 categorical factors among the farming practices hypothesized to have an influence on interactions with wild boars (9, 12, 15, 46). The selection criteria and categorical factors used for these variables are provided in Supplementary material S2.

#### 2.2.3 Study area and spatial scales

From an administrative point of view, Corsica is a region divided into two departments, “Haute-Corse” (Northern Corsica) and “Corse du Sud” (Southern Corsica). We collected data in two micro-regions in Southern Corsica, including “Haute Gravone” and “Secteur Ajaccio” and six micro-regions in Northern Corsica including “Cap Corse,” “Nebbiu,” “Balagne,” “Haute Corse Intérieure,” “Castagniccia” and “Plaine Orientale” (Supplementary material S1). The choice of these micro-regions was not only based on the possibility of collecting information from different geographic locations but also, on the possibility to test our approach on a representative and diverse sample of farming systems, land uses and vegetation types of the island.

In France, municipalities represent the smallest administrative level. Although not ideal because the land used by pigs does not always coincide with administrative boundaries, we considered the municipal scale to be the most practical and appropriate to represent the distribution of the pig population. The data collected on each pig farm was converted to the municipality scale on the basis of the ratio of the extent of land used by each herd in each municipality to the total area of land used by each herd. By combining this ratio with the size of the herd, we calculated the number of pigs in each herd in each municipality.

### 2.3 Exploring the diversity of pig farming systems in Corsica

The diversity of farming system based on reported practices was explored following three steps.

#### 2.3.1 Identification of clusters

The MCA was performed on 16 categorical factors (Supplementary material S2) describing the farming systems’ practices to summarize the information in a lower-dimensional Euclidean space (five dimensions) where distances represent similarity (16). Next, using Ward’s method, hierarchical clustering was performed of the MCA results to identify groups of farmers who use similar practices, subsequently termed “H-clusters.” Both operations were performed in R version 3.5.3 using the package FactoMineR for MCA (47) and HCPC (48). In the hierarchical clustering process, we considered the inertia gain ratio as the parameter determining the variance gain when the number of clusters increased.

#### 2.3.2 Validation and ranking of the clusters by local experts

A meeting was organized with a group of nine pig farming experts from different micro-regions to present our methodology and results and validate the conformity of our farm classification. The group was made up of four breeders and five technicians from pig farming -related organizations with a solid background knowledge of the Corsican pig sector, based on their activity, experience and training. During the meeting, we combined three types of participatory exercises selected for their complementarity (49) and their ability to capture and leverage local knowledge and expertise (50): focus-group discussion (51), cluster ranking, and proportional piling (32). The details on these participatory methods can be found in Supplementary material S3.

#### 2.3.3 Classification tree for cluster classification

We performed a classification tree (52), called “T-cluster,” with the Rpart R-package (53) to classify individuals not included in the initial HCPC analysis and farms identified in other data collection into specific clusters. We considered tree combinations of the different factors mentioned by experts as having the strongest impact on interactions and compared their percentage of correspondence with the HCPC attribution (called “H-cluster”). In this way, we selected trees with the least divergence between the “H-cluster” and “T-cluster” classifications.

### 2.4 Quantification of an index of potential interaction (IPI) per municipality

We selected the 155 municipalities for which we had sufficient data to characterize at least 50% of the farms, accounting for 144 farms and 21,807 pigs. To quantify the index of potential interaction (Y) due to pig farming based on its presence and practices in each municipality, we used a weighted average of the five cluster-specific pig-densities (X_1_ to X_5_):


$$Y=\sum w_{i}X_{i}$$

where the set of weights $w_{1},\ldots ,w_{5}$ , verify $\sum w_{i}=1$ determined by experts depended on whether the interaction Y is direct or indirect. The IPI could thus be interpreted as an effective number of animals at risk, and used to compare municipalities with different distributions of farming systems.

### 2.5 Characterization of municipalities

A principal component analysis (PCA) was performed on the number and density of pigs in each of the five clusters and on the surface area of the 155 municipalities included in the study (11 variables in total), to identify their main patterns of variation. The PCA provided a reduced representation of the profiles of municipalities on a two-dimensional Euclidean space, and allowed us to explore and investigate cases with different characteristics but similar levels of risk of interaction between domestic pigs and wild boars.

## 3 Results

### 3.1 Pig farm typology

Based on the results of the MCA, the distribution of dimensions explained 38.9% of the total variance with each dimension contributing to at least 5% of this value. In our case, evaluation of the inter-cluster inertia gain revealed that the best cut-off values were three and five. Based on this statistical evaluation and on our field observations, we chose five clusters (Supplementary material S4). All the variables in the analysis were identified as significant (*p*-value <10^−3^) except the period of domestic boar castration (*p* = 0.0068). Among the 103 farms studied, 16 were in H-cluster 1, 21 in H-cluster 2, 20 in H-cluster 3, 39 in H-cluster 4, and seven in H-cluster 5.

Table 1 summarizes the main characteristics of the five farm types identified. A detailed description is available in Supplementary material S5. Clusters 1 to 3 represented farms where pigs were allowed to free-range all or part of the year. Conversely, clusters 4 and 5 represented farms where pigs were fenced in all year round, either outdoors for cluster 4 or in a building for cluster 5. The main differences found between clusters 1 and 3 were in terms of free-ranging time, partial for cluster 3, and reproduction management, which was more controlled in cluster 2 than in cluster 1.

**Table 1:** Summary of the five types of farming practices used in the typology.

Cluster	Main breed	PDO	Farrow	Free-ranging	Fencing	Material used for fencing	Waste left outdoor	Feed supplement supplied	Spaying of sows	Mating in fenced area	Enclosure used for farrowing	Frequency of hybrid litters	Interactions with other pigs
1	Mixed or population	No	Yes	All year round	No fences or partial	Wire grid	Yes	No rules	No	No	No or yes	Regular	Yes
2	Nustrale	Yes	Yes	All year round	No fences or partial	Wire grid	Yes	Seasonal, some regularly	Yes or no	Yes	Yes	50% never, 50% sometimes	Yes
3	None	No	Yes	Seasonal (Autumn/ Winter +/− Summer)	Some fully fenced, some partial	Wire grid	Yes	Regularly	No or yes	Yes	Yes	Sometimes	Yes
4	Nustrale or mixed	Yes or No	Yes	Never	Fully fenced	Steel mesh or wire grid	50% yes 50% no	Regularly	No	Yes	Yes	Never	No
5	Other (LW, Duroc)	No	No	Never	Fully fenced	Construction	50% yes 50% no	Regularly	No	Yes	NC	NC	No

### 3.2 Use of local knowledge for validation of our farm typology and assessment of interactions between wild and domestic pigs

#### 3.2.1 Validation of farm typology using local knowledge

During focus group sessions, experts readily agreed on the definition and representativity of the five farming clusters of pig farming systems occurring in Corsica. When talking about the H-clusters and factors of interest, experts tended to focus on direct sexually driven interactions, but when considering a wider range of interactions, they drew their attention to three major factors, including sow reproduction, fence and carcass management:

– Reproductive Management: The key factor reported in order to minimize sexually driven interactions was the availability of receptive reproductive females in free ranging systems. The experts considered spatial compartmentalization of reproductive females and surgical neutering of animals not intended for reproduction as the two major strategies likely to have a positive impact in reducing the attraction of wild boars toward farmed sows and hence potential interactions.
– Fence management: the experts emphasized the fencing material used and its maintenance were the two major limiting factors for impeding wild boar incursions. In their opinion, only building welded mesh and electric fences, although not perfect, under regular maintenance could potentially contain wild boar incursions in the farm and prevent interactions with their domestic pigs. The use of adequate and well-maintained materials was considered instrumental to avoid spaying females non-targeted for reproduction. In all other cases, the absence of spaying necessarily led to incursions and subsequent interactions, particularly sexually driven ones.
– Management of carcasses and offal: experts regretted in Corsica this aspect was overlooked and becoming an increasing concern because some parts of pig carcasses are no longer processed and wild boar meat is less frequently consumed by hunters.

Concerning free ranging systems, experts identified a strong influence of the season during which animals were widely kept free ranging (autumn) in the number and length of interactions.

This local knowledge and expertise enabled us to refine our classification and agree on a final cluster typology concerning zootechnical practices.

#### 3.2.2 Final T-cluster classification

**Figure 2:**
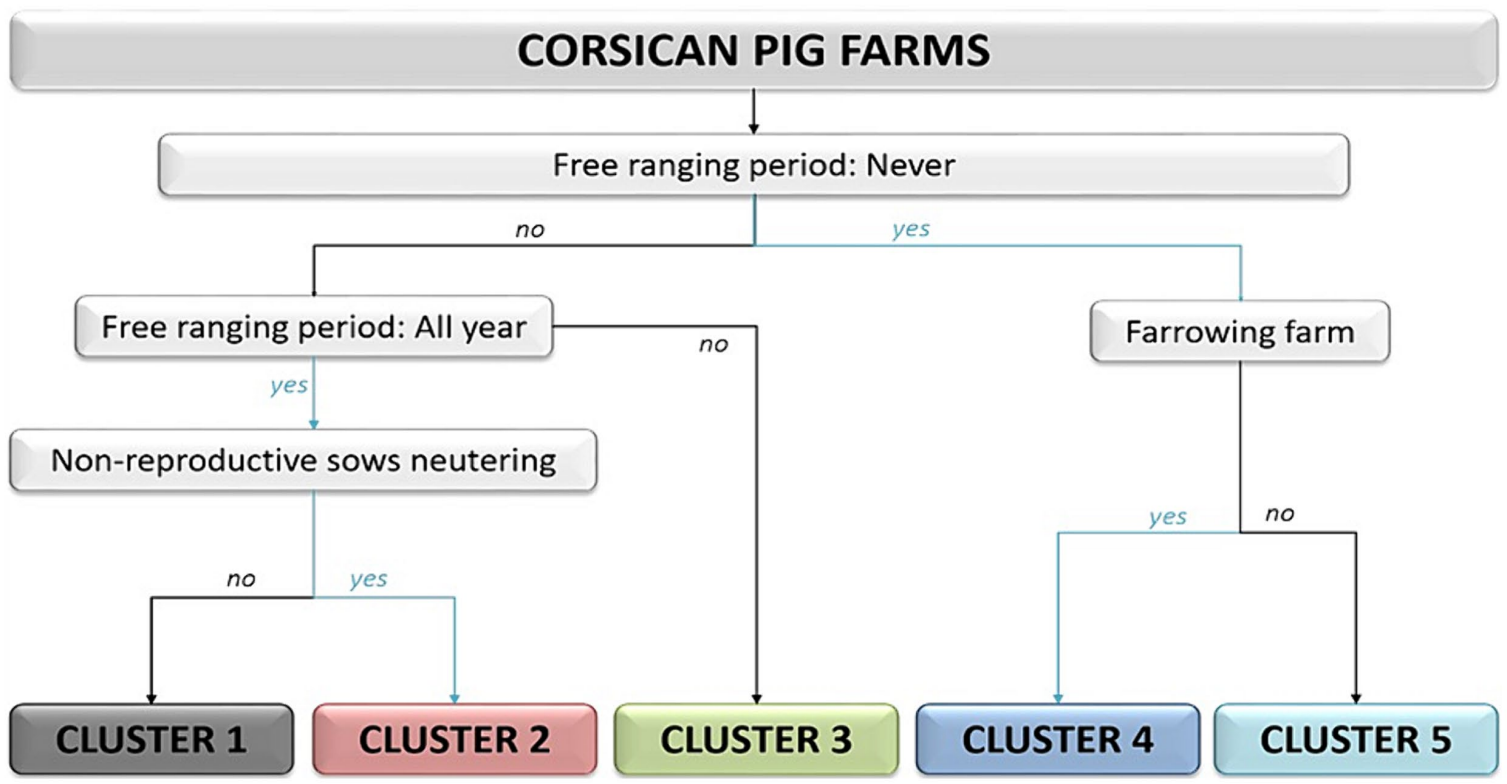
Final cluster classification combining typology and validation of local experts. These five clusters represent the T clusters.

The selected tree based on the above-mentioned criteria included 19 results that diverged from the results of the HCPC of the total 103 farms (Figure 2). Such rate was considered to be acceptable given that the number of divergences most frequently observed for trees obtained with Rpart was 15 out of 103. Moreover, unlike the original tree (9/15 cases), our designed tree respected the precautionary principle in 17 out of 19 cases, meaning that, in the event of divergence, the farms were classified in a higher-risk T-cluster than the original H-cluster. Hereafter, all the results presented are based on the T-cluster classification.

**Figure 3:**
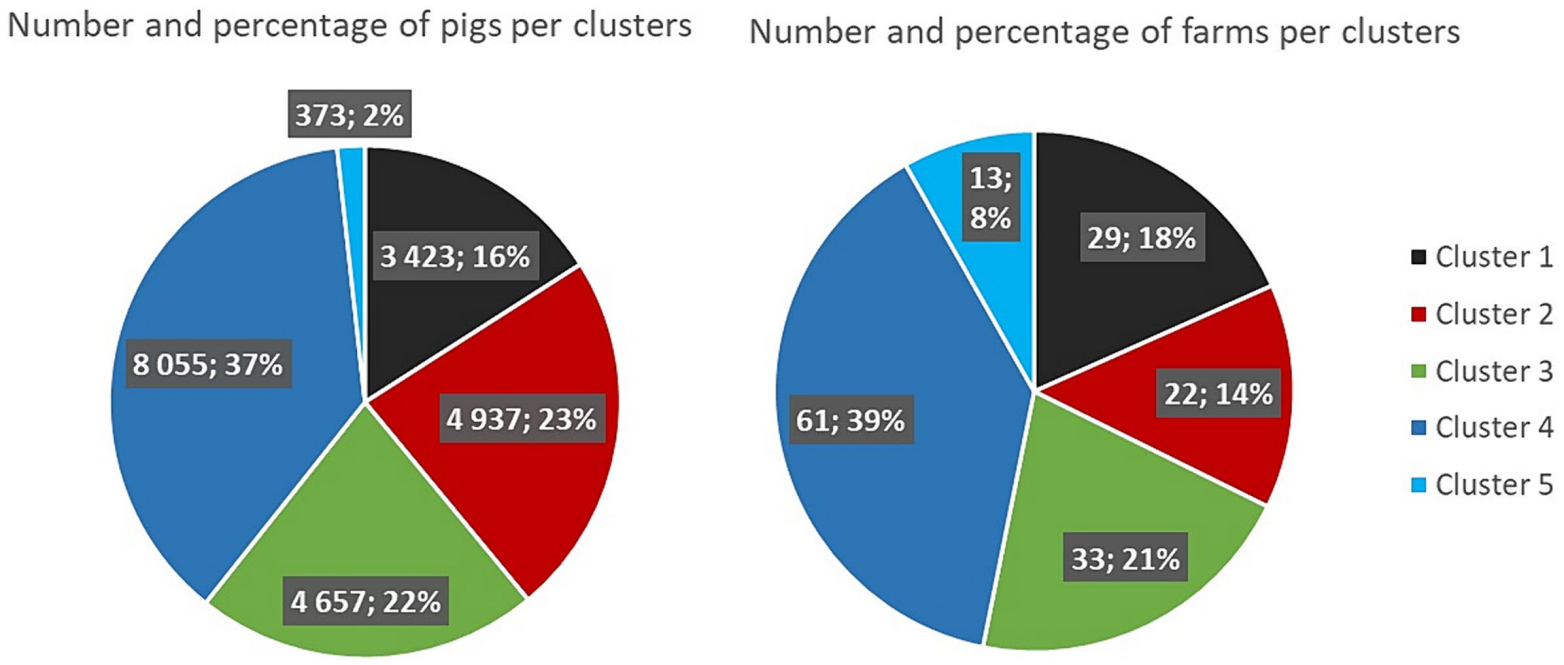
Distribution of number of pigs and farms in each cluster (T Clusters). Clusters 1, 3 and 4 show a certain homogeneity between the number of farms and the number of pigs in each cluster. The difference in percentage between number of farms and number of pigs for clusters 2 and 5 indicates a higher average farm size than clusters 1, 3, and 4.

As shown in Figure 3, cluster 4 is the biggest, representing 37% of the pig population, whereas cluster 5 only represents 2%; clusters 2 and 3 represent equivalent proportions (23 and 22%) and cluster 1 represented 16% of the pig population. Although not all the municipalities in the selected micro-regions were accounted for, coverage of the main breeding micro-regions of our selection, Castagniccia and Haute-Gravone, was almost complete.

#### 3.2.3 Weighting of the index of potential interaction (IPI) for each cluster

A consensus on the ranking of H-clusters was easily reached by the focus groups on the case of direct interactions. However, the concept of indirect interactions required more discussion to reach a consensus on ranking results. The results of the focus group on the main factors defining clusters (see 1.2) were subsequently confirmed in the discussions concerning ranking.

Concerning direct interactions (Table 2), higher numerical values were revealed for clusters 1 and 3 than for the other clusters. The main factors that influenced ranking and piling by experts were reproductive management, spaying of sows, and seasonality of free ranging animals. Moreover, although cluster 3 had less probability of interactions occurring during the free-ranging period than cluster 2, experts agreed that spaying of sows non targeted for reproduction was the most important factor influencing direct interactions, which explains why cluster 3 was considered to have more direct interactions than cluster 2.

**Table 2:** Weighting and ranking of clusters by local experts based on the occurrence of direct and indirect interactions in pig farms.

Rank (1 = highest)	Cluster	Main arguments for ranking (compared with a lower-ranked cluster)	Numerical weight of the IPI (mean of expert’s results)
Direct interactions			
1	Cluster 1	No management of reproduction Spaying of sows not intended for reproduction: none Pigs allowed to range free all year round	43.7
2	Cluster 3	Management of reproduction Spaying rate of sows not intended for reproduction: 35.0% Free ranging part of the year	29.5
3	Cluster 2	Spaying rate of sows not intended for reproduction: 57.1%	16.5
4	Cluster 4	No free ranging	7.4
5	Cluster 5	Type of fencing	2.9
Indirect interactions			
1	Cluster 1	Free ranging all year round Carcasses and leftovers left on the ground outdoors	36.8
2	Cluster 2	Better management of reproduction: reduced attraction of wild boars	31.2
3	Cluster 3	Free ranging part of the year	19.6
4	Cluster 4	No free ranging = very few resources shared Better management of carcasses and leftovers	7.4
5	Cluster 5		5

Concerning indirect interactions (Table 2), two experts disagreed with the order proposed in the proportional piling ranking exercise. In their opinion, clusters 1 and 2 had the same weight because free ranging and waste management practices had a similar impact on interactions. However, they all agreed that free ranging facilitated the sharing of food resources such as pastures (but also carcasses or offal) and water points. This explained the lower value for cluster 3 and the similar values for clusters 4 and 5.

### 3.3 Data visualization

#### 3.3.1 The spatial distribution of pigs and types of practices

**Figure 4:**
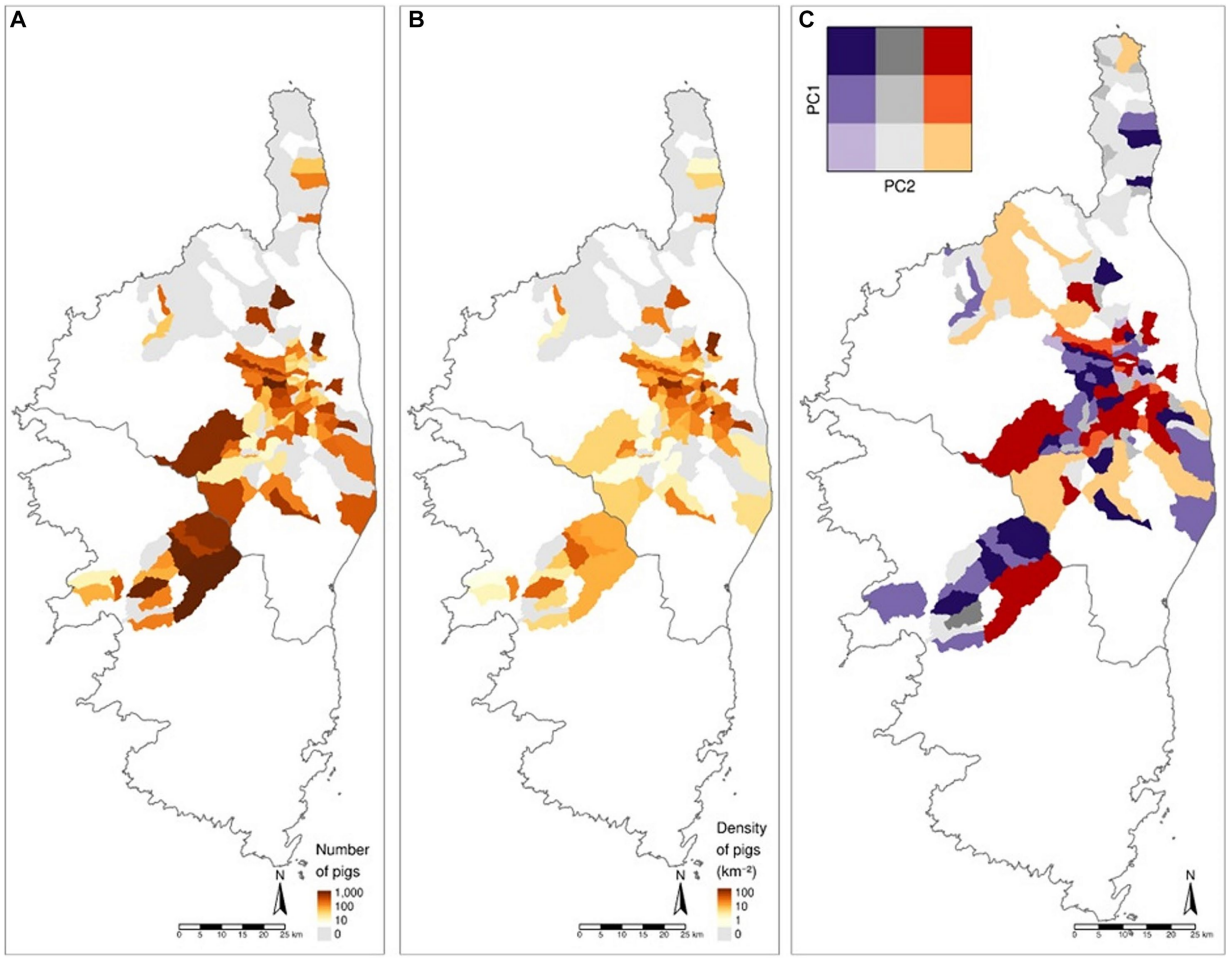
Maps of the number **(A)** and density **(B)** of pigs per municipality investigated. There are higher concentrations of pigs in the Haute Gravone and Castagniccia micro-regions. **(C)** Map of profiles of municipalities according to the principal components 1 and 2. Purple shades show a predominance of cluster 4 or 5 farms, while red shades show a predominance of cluster 1 or 2 farms.

Densities of pigs and absolute numbers were closely correlated and independently of the area in the first PCA factorial plane, meaning that high densities tend to be explained by large numbers rather than by small surface areas. In contrast, the density of pigs in cluster 3 was explained to a large extent by smaller areas. Density thus enabled an accurate representation of the number of pigs at the scale of the island. A number of municipalities could be distinguished by the number of pigs, especially in the case of Castagniccia micro-region (Figures 4A,B).

The different types of farming practices were distributed across the island, even if some municipalities stood out among the dominant clusters. Clusters 1 and 2, characterized by year-round free ranging, were more frequent in mountain and piedmont municipalities (Castagniccia, Plaine Orientale), while Clusters 3 and 4 were found in most regions, but specially in municipalities with high pig densities, such as Castagniccia and Haute-Gravone. Clusters 3 and 4 were dominant in the Haute-Gravone, whereas fewer farms mainly corresponding to 4 and 5 cluster types, were located in Cap Corse.

The PCA results showed that 42% of the variability of densities and number of pigs in each cluster and the surface area of municipalities can be explained in two dimensions. The first principal component (PC1) measured volume (i.e., number and density of pigs regardless of the cluster), while the second component (PC2) measured specificity (i.e., concentration either in clusters 1 and 2 or in clusters 4 and 5). The municipalities could be reasonably well characterized using only two quantitative variables, such as the densities of pigs in clusters 1–2 and 4–5, or equivalently, principal components one and two. The different colors in Figure 4C identified municipalities with similar profiles in terms of cluster distribution. The visible contrast between different aggregations of municipalities suggests a neighborhood effect or the use of similar practices depending on the characteristics of the area, such as valleys as opposed to mountains. This effect is particularly relevant in Haute-Gravone and in the western part of Castagniccia where clusters 4 and 5 were predominant, while in the central part of Castagniccia, profiles of municipalities were primarily composed of clusters 1 and 2.

#### 3.3.2 The spatial distribution of the IPI based on pig farming practices

**Figure 5:**
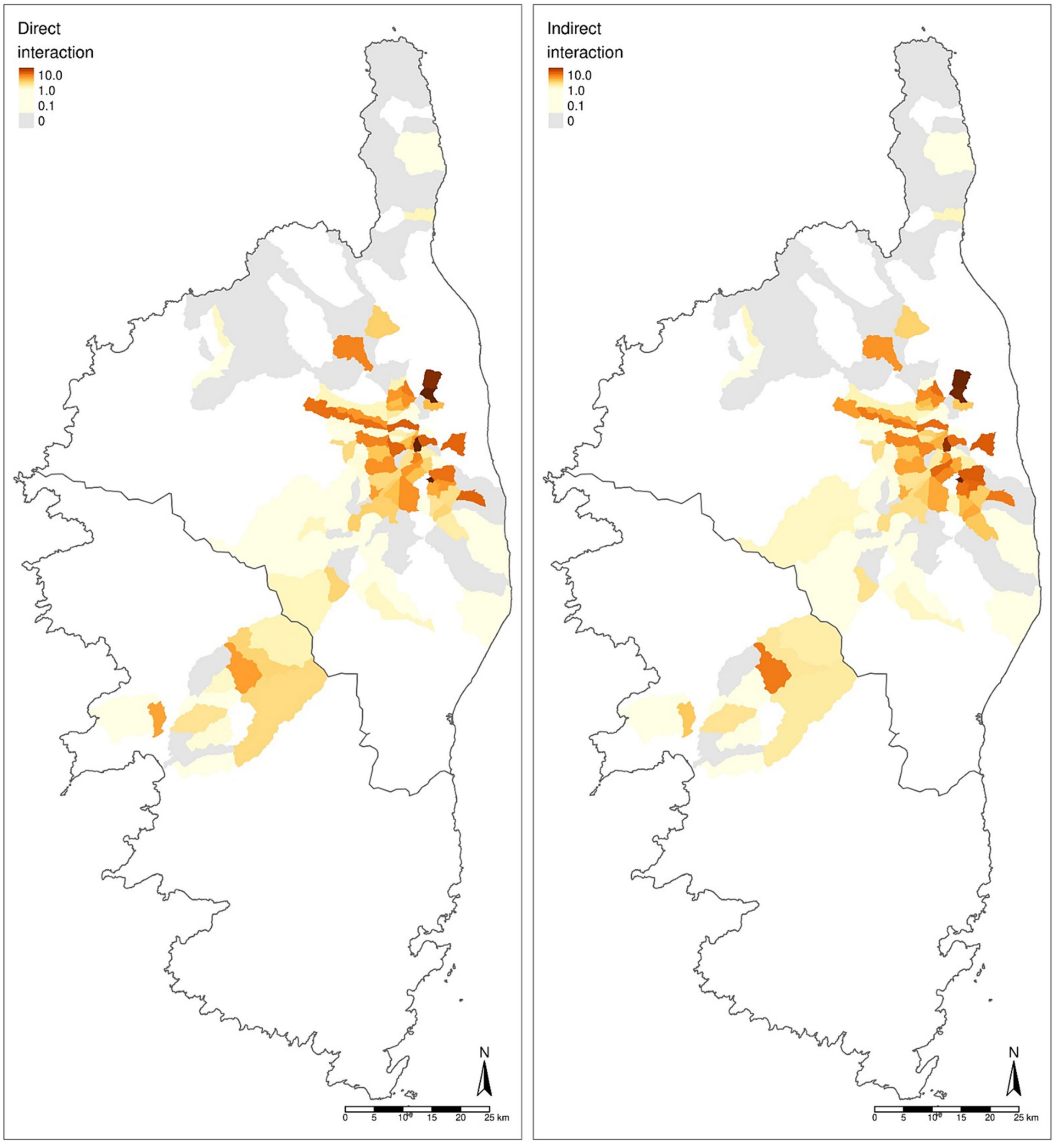
Map of IPI based on potential direct and indirect interactions as estimated by the custom weighted averages of cluster densities.

In general, direct and indirect IPI co-evolved within municipalities as shown in Figure 5. The spatial projection (logarithmic scale) of these indexes per municipality made it possible to qualify them according to their IPI and to distinguish hotspots of potential interaction between domestic pigs and wild boars.

These hotspots mainly concern municipalities or groups of municipalities with both a high density of farms and number of pigs raised in them. A major hotspot was located in the north central part of Castagniccia, which also showed a more variable IPI than other more homogeneous microregions such as Haute-Gravone.

#### 3.3.3 Heterogeneous farm profiles despite similar IPI

In our results IPI seemed to correlate roughly with pig density. However, farming practices, through cluster attribution, could have had a significant influence in IPI values. For example, the municipality of Lano encompassed 315 pigs raised on a cluster 4 farm and although the density of pigs was high (61.3 pigs/km^2^), the IPI remained relatively low (9.04).

Figure 5 showed that interactions varied from one municipality to another. In this case, IPI alone was not sufficient to qualify the interaction as some municipalities had similar IPI values, but different configuration in terms of clusters and thus, of practices (Supplementary material S6). Thus, the map shown in Figure 5 allowed the identification of hotspots of high interaction risk in pig farms despite the municipality cluster distribution profile concerned provided a better understanding of the parameters that could influence interactions.

## 4 Discussion

### 4.1 Outlining the diversity of farmers’ practices to assess the interaction

Research to understand wildlife-livestock interfaces has gained increasing attention in the last decades (54). Such interfaces represent complex and dynamic socio-ecological systems influenced by several components including pathogens, hosts and human behavior. Considering that the environment and land use can influence human activities and that pig farming practices can have a major impact on wild boar-domestic pig interactions, our work proposes a territorial large-scale approach to provide an index of the risk of interactions based on predominant pig farming practices. The advantage of our approach is that it allows the development of different options for the management of infectious interactions from a regional policy perspective (identification of hotspots), as well as from a farming system perspective (major drivers of interactions in pig farming practices). Finally, the spatialization of our results required making choices at the administrative level (municipality scale) that can be useful for decision making in the management of shared infectious diseases affecting the pig industry or public health.

As based on previous literature (29, 41), a farming practice is not merely a simple technical choice disconnected from the farmer’s overall logic. A specific practice is often linked to the implementation of other practices used to pursue the same goal. For example, in our study, farmers who wanted to avoid wild boar intrusions in their farm could choose between improving biosecurity by building a fence to, spaying all young sows not intended for reproduction, or a combination of both methods. The selected clusters managed to capture this diversity of practices targeting the same goal rather than a series of disconnected practices. However, in our analysis, the division of practices in clusters shaped by key practices could have hidden the overall logic of farmers’ choices. Farmers’ logic is better considered using the “systems of practice” concept highlighted by some pioneering work on rural sociology from last century (55). More recent publications suggested that the “systems of practice” approach allows a better understanding of farms complexity in a region as it connects the object of the study (here biosecurity) to other dimensions of farming systems and to farmers’ overall logics (56). Hence in our study, farmers identified seasonal feed resources and reproduction as major drivers toward which their practices should be targeted to manage individual risk. One possible explanation is that in autumn, the availability of abundant resources such as chestnuts and acorns, coincides with the rutting period of adult wild boars, and the period of oestrus period sows. Autumn thus represents the most suitable moment of the year for the occurrence of direct interaction. This may explain the importance of reproduction practices underlined by pig farmers in contrast to observations reported in drier Mediterranean areas, where sources of water in spring and summer appear as predominant drivers of interaction (18). An originality of our approach is that the quantification of our proxy of interaction (IPI) is not based on biosecurity measures and standards as in other studies, but rather on the farmers’ perception of the local drivers and available methods to address the problem. In this case, spaying sows non-targeted for reproduction, despite being discouraged by animal health professionals and questionable in terms of animal welfare, is frequently practiced in Corsica and perceived as an important factor for mitigating sexually driven interactions with wild boars (16, 17, 25).

Several authors have underlined the advantage of using clustering to process local knowledge collected by experts (57, 58) as well as organizing the information in systems based on their statements and/or opposing perceptions (59). Cluster analysis is always a representation of reality in response to a specific research question (in this case pig farming practices). Other attempts to analyze and classify pig farming in Corsica produced different representations of the same reality based on different zootechnical, sanitary or socio-economic perspectives (16, 60, 61), the typology of Relun et al. being the closest to our results. Comparable pig farming adaptations can be found in other Mediterranean islands or regions (61) with in some cases, an accepted level of risk, as illustrated by regular outbreaks of African swine fever reported for decades in neighboring Sardinia (5, 22, 62). Clusters 2 and 3 can be distinguished from cluster 1 by their implementation of more restrictive practices in terms of domestic pig-related behavior and its interactions with wild boar. In the opinion of the local experts, cluster classification could be influenced by the priority given by each farmer to avoid direct (for cluster 2) or indirect (for cluster 3) interactions. These practices were common, and often associated with the occupation of agriculturally abandoned areas. Finally, the remaining typologies included more standard clusters in which, the spatial behavior of animals was moderately (cluster 4) or strongly (cluster 5) constrained. Cluster 4 allowed complete control of the herd, avoiding conflicts between neighbors, making better use of feed resources, while being compatible with origin certification (PDO). It also permitted the combination of biosecurity measures (control of contact with wild boars) with a semi-extensive free ranging system.

### 4.2 Methodological contributions and limitations

The choice of participatory methods allowed us to deal with two main challenges. On one hand, the information gathered compensated for the lack of an official exhaustive and updated list of farmers, a situation which is quite common in some regions or countries and represents a constraint. On the other hand, the contribution of key informants compensated for other difficulties in the data collection process such as access to isolated farms, the absence of a reliable and complete list of available farmers, missing data or the potential lack of farmers willing to participate. Although it is possibly not exhaustive, the list of farmers we were able to compile through the implementation of our methods was considered by several extension agents and health services to be the most complete list of farmers obtained to date. Moreover, our approach made it possible to cross-reference the information gathered from different informants in the same area. In addition to their own zootechnical know-how, key informant farmers provided information about their immediate neighborhood as represented by their informal network of local stakeholders. Despite there was a risk of subjectivity in the information provided by key informants this bias was compensated by triangulating information from different farms in the same region. Moreover, the way we collected our data shed some light on the problems experienced by local actors in their respective situations (63). The role of local experts as additional providers of local knowledge, represents another innovative aspect of our approach and more generally, demonstrates the relevance of applying participatory methods in such contexts (64). Such an approach fell somewhere between two standardized methods such as the expert elicitation and stakeholder opinion survey. As an expert elicitation process, their choice could be considered subjective because it was mainly based on the local social recognition based on their pig farming experience. As social stakeholder survey, the sample size (*n* = 9), was below the minimum required threshold. Nevertheless, despite these recognized methodology flaws, the combined adaptation of these two approaches succeeded in collecting relevant information for the purpose of the study.

A strong assumption made in our approach was that the probability of interaction was mainly driven by pig farming practices rather than by the distribution and abundance of wild boar populations. The local abundance of wild boar populations is likely to influence the occurrence and frequency of wild boar incursions into low biosecurity farms and thus, the occurrence of interactions with domestic pigs (9). However, since information on wild boar abundance at the scale of the island of Corsica is not available, our spatial representation of the IPIs only considered the pig farming perspective and not the influence of wild boar population abundance. Therefore, the resulting map is provisional first assessment to this topic and needs to be completed with information on wild boar estimated densities and compared with other field data such as genetic introgression of wild boar populations (65) or the distribution of shared swine pathogens (8, 11, 15). Spatializing our index in order to successfully link the information obtained through our participative approach with the development of strategies to manage disease risks in our study sites is a major challenge and goal in the Corsican context that relatively few studies have addressed in the literature to date (18, 44). One of the first difficulties encountered when addressing this challenge was the choice of an adequate spatial scale to map the risk of interaction. By choosing the municipality scale, we sacrificed precision and representativeness. However, neighborhood effects between farms represented an unavoidable bias. In addition, in the case of small-sized municipalities, farming estates could exceed the boundaries of a single municipality and often, the pigs were not equally distributed but rather concentrated in parts of the municipality which were more resource-abundant. Another source of bias occurred when the surface or perimeter of a farm was located between two municipalities or in a different administrative division from the one it was registered.

The spatial distribution of clusters contributed to identify contrasted micro-regions that could be informative from the risk management perspective. Indeed, calculating a proxy of wild-domestic pig interaction in pig farming areas such as IPI enables the identification of regional “hotspots,” and the municipalities profile in terms of cluster distribution provides key information to target pig farming development and biosecurity efforts. Last but not least, our equation to calculate the IPI overemphasized pig density in detriment of farmers practices. This limitation needs to be addressed in future work, particularly when considering municipalities that host a wide diversity of farming systems.

## 5 Conclusion

Our work proposes an original methodology to collect zootechnical information and classify pig farms in order to spatially represent and compare a proxy of interaction with wild boars among 8 pig farming micro-regions from the Corsican territory. Our approach was particularly successful to identify some micro-regions particularly prone to extensive pig farming, as potential hot spots of interaction with wild boars. The method is based in the combination of approaches from different disciplinary fields including social sciences, epidemiology, animal husbandry, geography and ecology. This preliminary information could help to identify priority areas for the implementation of regionally-adapted management strategies of porcine disease shared with wild or feral pigs. Our approach is particularly applicable regions prone to extensive livestock farming where information on farming practices is lacking. Our method has the potential to be improved and implemented at a larger territorial scale not only in Corsica but also in other regions confronted with similar types of extensive animal production, exposed to interactions with wildlife and challenges of disease transmission risks.

## Data availability statement

The raw data supporting the conclusions of this article will be made available by the authors, without undue reservation.

## Author contributions

FJ, FCh, FCa, ML, and LD contributed to conception of the study. LD designed method, implemented data collection, organized the database and performed the statistical analysis, and wrote the first draft of the manuscript. FM contributed to the statistical analysis. FJ and FCh contributed to the supervision of the study. FJ, FCa, BT, and FCh completed and improved different sections of the manuscript. All authors contributed to the manuscript revision, read, and approved the submitted version.

## Supplementary material

### SUPPLEMENTARY FIGURE 1

Map of the micro-regions of Corsica.

### SUPPLEMENTARY FIGURE 2

Factor maps of the 5 clusters from the MCA.

### SUPPLEMENTARY FIGURE 3

Comparison of profiles of villages with the same IPI values.
