# Supplementary material for: A combined methodological approach to characterize pig farming and its influence on the occurrence of interactions between wild boars and domestic pigs in Corsican micro-regions
Source: Front Vet Sci. 2024 Apr 2;11:1253060. doi: 10.3389/fvets.2024.1253060 (PMC11019438; doi:10.3389/fvets.2024.1253060)
Supplement: Supplementary file 1 [file Data_Sheet_1.PDF]

Supplementary Material

A combined methodological approach to characterize pig farming and its influence on the occurrence of interactions between wild boars and domestic pigs in Corsican micro-regions.

Dupon, L.*, Trabucco, B., Muñoz, F., Casabianca, F., Charrier, F., Laval, M. and Jori, F.

*** Correspondence:**
Liane DUPON
dupon.liane@gmail.com

S1: Materials and method – Map of SNRA

Figure S1: Map of the Aggregated Small Natural Regions in Corsica (French acronym PRNA)

S2: Materials and methods – Selection of farm practices

Among the factors that matched our criteria, we selected those that described agricultural practices, were readily available and whose categories were easy to define. We performed a descriptive analysis of the initial data to identify the main categories (listed in the table S2) for each factor. Seven other factors (in grey) were added as supplementary variables because of lack of respondent (e.g. concerning the period of castration of males not intended for reproduction), lack of diversity in the responses (e.g. concerning the castration of males not intended for reproduction), being useless for our analysis (e.g. micro-region) or because the variable was a consequence rather than a cause of interaction (e.g. frequency of hybrid litter). These supplementary variables were not involved in the determination of the components and therefore in the analysis but can be projected on these components. Concerning the number of pigs, to facilitate MCA and descriptive analysis, we defined four categories based on the empirical distribution of our data. We consequently classified farms based on the number of pigs: 0-90 pigs: small, 91-199 pigs: medium, 200-399 pigs: large, more than 400 pigs: very large.

Table S2: Factors selected for the statistical analysis

Characteristics of farms	General practices	Management of reproduction	Interactions
Aggregated small natural regions *(micro-region)*	**Free ranging** *(All year round/Part of the year/Never)*	**Spaying of sows not intended for reproduction** *(YES/NO)*	**Interaction with pigs from other farms** *(YES/NO)*
Breed *(Nustrale, Corsican type, Crossbred, Other selected breeds)*	**Period of free ranging** *(All Year/ Autumn & Winter / Summer & Autumn & Winter/ Never)*	**Castration of males not intended for reproduction** *(YES/NO)*	**Frequency of hybrid litter** *(Regular /Sometimes /Never)*
Size of the herd *(Small/ Medium/ Large/ Very large)*	**Fencing** *(No fence/ Fully fenced/ Partially fenced)*	**Time of castration in males' lifespan** *(Early/ Late/ Not specified)*	**Management of these litters** *(Culled, raised)*
PDO certification *(YES/NO)*	**Tight fence** *(YES/NO)*	**Mating in un-fenced area** *(YES/NO)*	
Farrow-to-finish pig farm *(YES/NO)*	**Fencing material** *(Wire grid/ Steel mesh/ Electric/ Constuction)*	**Specific fenced area for mating** *(YES/NO)*	
	**Supply of supplementary feed** *(Regularly/Seasonal/Never)*	**Specific fenced area for farrowing** *(YES/NO)*	
	**Period of supplementary feed supply** *(All year round/Summer/Spring & Summer & Autumn)*	**All sows can enter these areas** *(YES/NO)*	
	**Carcass and offal disposal** *(YES/NO)*		

S3: Materials and methods – Definition of clusters using a participatory approach with the group of experts

Each cluster defined in this work represents a type of farming involving practices that differ from those used in the other clusters. Since these practices have different consequences for the

interaction, we hypothesised that each cluster corresponds to a different degree of interaction. To be able to map and to compare results from one micro-region to another, we needed to systematize our method by assigning numerical values to the different degrees of interaction. Since no data are available in the literature on these topics, we decided to work with a group of local experts using three different types of activity:

- Focus groups: To facilitate discussion, we wrote a discussion guide as a framework for the moderator and a few slides to present the notion of interaction, the practices we were considering in our study, and the clusters. Each expert had at his/her disposal a document summarising the data mentioned in the presentation. Rather than answering questions, group members were encouraged to talk to one another to share ideas and to comment on other experts' experience. The main objectives of this exercise were to compare our work with the reality of the field and to identify practices that influenced the definition of our clusters.

- Ranking: To facilitate the exchange of information and to ensure all views would be expressed, we divided the experts into two groups. Once the two groups joined forces again, their results were compared and discussed until a consensus was reached on the classification of clusters concerning both direct and indirect interactions.

- Proportional piling: On a blank document, we printed five equal rectangles representing the different clusters. The experts had to distribute 100 tokens in the rectangles proportionally to the degree of interaction they assumed for each cluster. We then calculated the mean of their results to determine the weight to attribute to each cluster.

S4: Results – MCA results for the determination of the clusters

The MCA method uses each individual's representation according to their categories in a Euclidian space defined by specific dimensions. The purpose of these dimensions (also called components or projections) is to capture the variance of variables as efficiently as possible. The dimensions are thus linear combinations of the variables studied, which represent different amounts of variance (characterised by eigenvalues) and are defined such that each component captures the variance not described by the previous one. MCA can be interpreted as orthogonal decomposition of the variance (also called inertia) of a data table.

The variables that most affected the five factors were free-range management, construction and maintenance of the fences and reproductive management of sows. Factor 1 was mainly influenced by free-ranging variables (whether the pigs ranged freely or not, the length of the free-range period), management of fences (presence of fences, tight fences or not, the material used to build the fences) and management of reproduction (was the farmer a breeder or not, were specific fenced areas used for mating and farrowing or not). Factor 2 was more linked to management of reproduction (was the farmer a breeder or not, did mating take place in a fenced area or not, were specific fenced areas used for mating and farrowing or not). Factor 3 was most influenced by variables concerning feed management (supplementation or not, period of supplementation), breed and PDO certification. Like factor 1, factor

4 was largely influenced by fence management and free-ranging variables. Factor 5 was very mixed as it was influenced by several variables.

Figure S4: Factorial map of the different clusters

S5: Results – Description of the five types of farming practices used in the typology

H-cluster 1 only included farms that practice free ranging all year round (87.5%) or during part of the year (12.5%). These farms had no fences (50%) or were only partially fenced or inefficiently (50%) fenced. Mating did not take place in fenced areas (75%) and in 25% of the cases, neither does farrowing. None of the farms in this cluster castrated or spayed animals non-targeted for reproduction. The great majority of these farms (93.7%) did not participate to PDO certification. All the farms owning pig herds that were irregularly fed were found in this cluster. Furthermore, these farms were identified as using some practices that are representative of a low level of management like late castration of males or raising hybrid litters (43.7% of farmers in the cluster). In addition, on the farms classified under this cluster (10/16), pig carcasses and carcass offal were left unattended and not disposed of. Finally, farms in this cluster regularly reported experiencing hybrid litters (75%), an indicator of sexual interactions between domestic sows and wild boars.

In H-cluster 2, all the farms practiced free ranging of pigs all year round (100%). These farms had no fences (28.6%) or were only partially fenced (71.4%) meaning, in both cases, that their fences were permeable. Pigs on these farms thus shared the same space with pigs from other farms. However, these farmers did control reproduction: mating took place in fenced areas (100%), farrowing in specific areas (95.2%) and 57.1% of these farmers spayed females non-targeted for reproduction. These farms mainly raised the “Nustrale” breed (85.7%) and used PDO certification. Concerning feeding practices, 66.7% of the farms in this cluster provided seasonal feed supplements, and 80.9% of the farmers left carcasses and leftovers outdoors. Finally, practices reported in this cluster seemed to be linked with medium size farms (91-199 pigs).

H-cluster 3 mainly encompassed farmers who keep their pigs in fenced areas and practiced free ranging of pigs only a part of the year (80%), in most cases, only in autumn and winter (62.5%) while others also left their pigs free in pastures in summer (30%). Accordingly, most of the pigs in these farms can interact with pigs belonging to other neighboring farms. Most of the farms (60%) used wire grid fences, if they did fence in their pigs. Most of the farmers in this cluster (85%) were not involved in PDO certification. Concerning management of reproduction, all the farmers (100%) had concentrated mating pigs in a fenced area and in 95% of the cases, farrowing also took place in specific areas. Moreover, 35% of these farmers neutered pigs not intended for reproduction. The majority of farmers (70.0%) in this cluster reported problems with hybrid litters, but infrequently.

In H-cluster 4, pigs never ranged free (97.4%). Pigs were kept in fenced enclosures within plots of different sizes. The majority (56.4%) were tightly fenced and the pigs consequently had no contact with other pigs. All the farms that used steel mesh fences were in this cluster. As the pigs never left the

enclosure, all the farmers in this cluster fed them every day all year round (100%). Mating almost always (97.4%) took place in fenced area which were not always specifically aimed for reproduction. Only 12.8% of the farmers spayed sows not intended for reproduction. Most of the farmers (87.5%) who did not leave carcasses and waste lying around outside were in this cluster. The proportion of farmers who never recorded hybrid litter is higher than in other clusters (51.3%).

Finally, H-cluster 5 grouped farmers who never practiced free range farming (85.7%). The pigs were kept indoors and, in most cases (71.4%) the enclosures were considered to be efficient. As none of these farmers were breeders, they were not concerned by management of reproduction, nevertheless 28.6% of them spayed their sows. Most of these farms (85.7%) were small (fewer than 90 pigs) and more than half (57.1%) reared non-Corsican selected breeds (Duroc and Large White among others).

S6: Results – Comparison of municipalities with similar levels of interaction

The cluster profiles in the municipalities differed despite having similar indexes of potential interaction. For example, with equivalent pig density, the municipalities of Rapaggio (density = 151.48 pigs/km^2), and Loreto (density = 129.21 pigs/km^2) had similar degrees of interaction, respectively 74.41 and 77.79. However, Rapaggio had 349 pigs, all of which were raised in cluster 3 farms, while the municipality of Loreto had 1 052 pigs distributed in clusters 1 (n=370), 2 (n=672) and 4 (n=10). The municipalities of Ucciani and Novale also shared a similar degree of interaction (15.01 and 15.25 respectively) but Ucciani had 941 pigs distributed among clusters 1 (40), 2 (700), 3 (105), 4 (60), 5 (36) giving a total density of 32.8 pigs/km^2 whereas Novale had 158 pigs all reared in cluster 2 farms at a density of 31.9 pigs/km^2. The municipalities of Bastelica and Bustanico had similar degrees of interaction (5.22 and 5.4 respectively). Bastelica had 1 214 pigs distributed among clusters 1 (220), 2 (24), 3 (950), 4 (20), at a density of 9.51 pigs/km^2. Bustanico had 186 pigs distributed among clusters 1 (36), 3 (50), 5 (100), at a density of 16.04 pigs/km^2.

Figure S6: Comparison of municipalities with similar levels of interaction
