# Supplementary figures and images for: A combined methodological approach to characterize pig farming and its influence on the occurrence of interactions between wild boars and domestic pigs in Corsican micro-regions
Source: Front Vet Sci. 2024 Apr 2;11:1253060. doi: 10.3389/fvets.2024.1253060 (PMC11019438; doi:10.3389/fvets.2024.1253060)

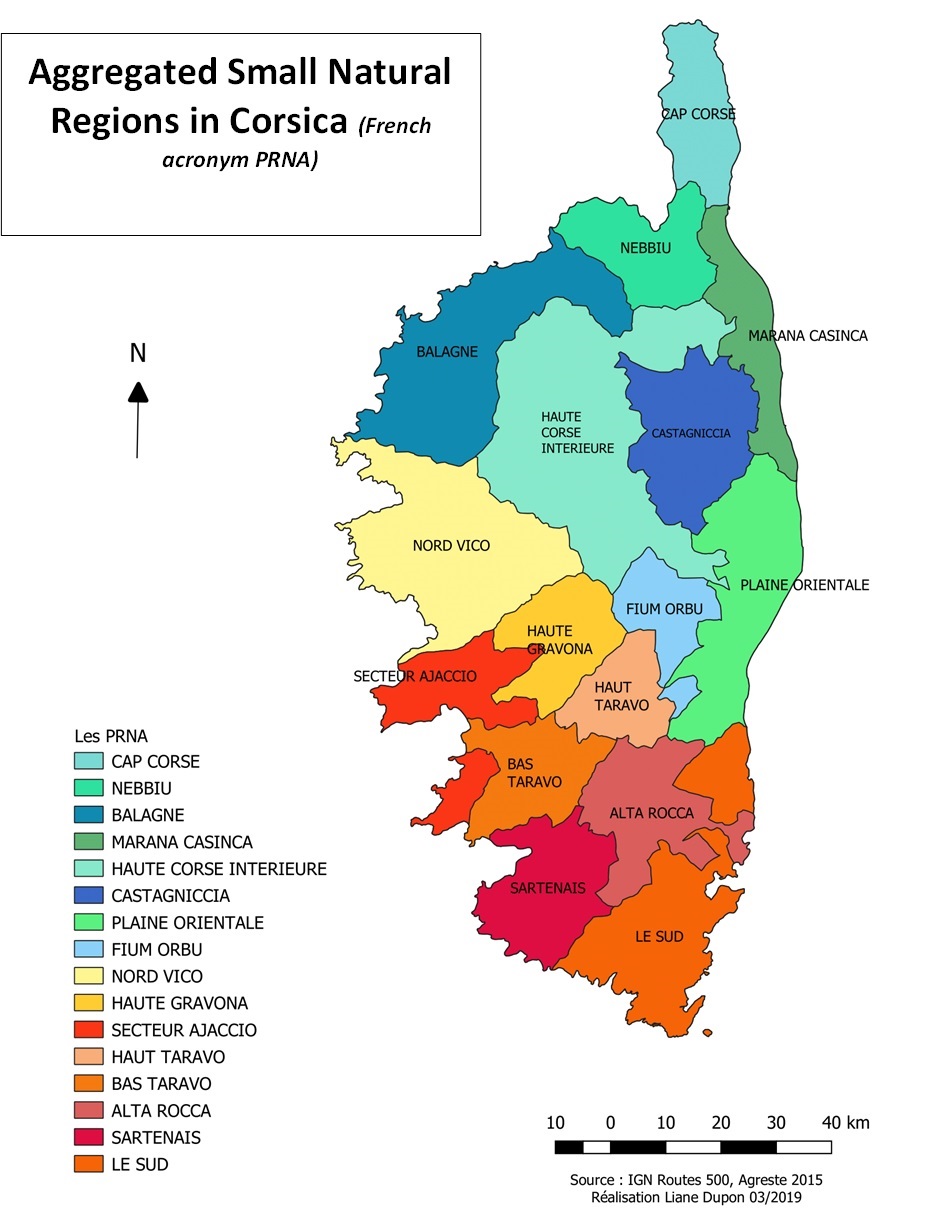

Supplement: Supplementary file 2 [file Image_1.JPEG]

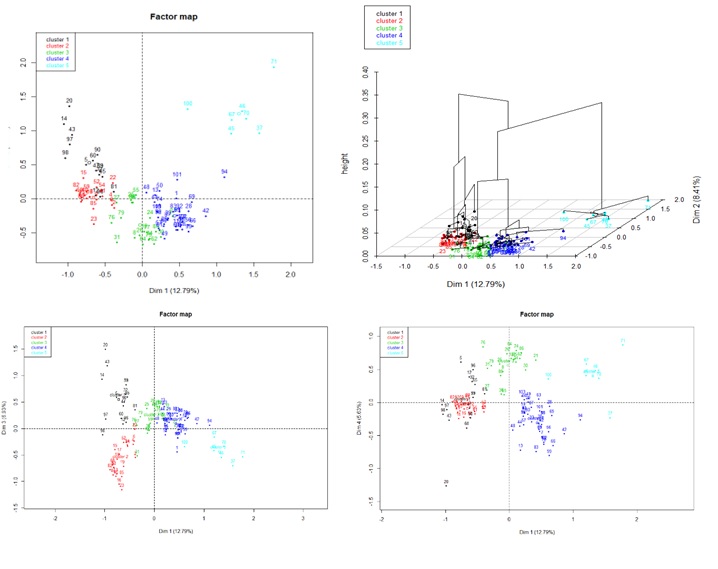

Supplement: Supplementary file 3 [file Image_2.JPEG]

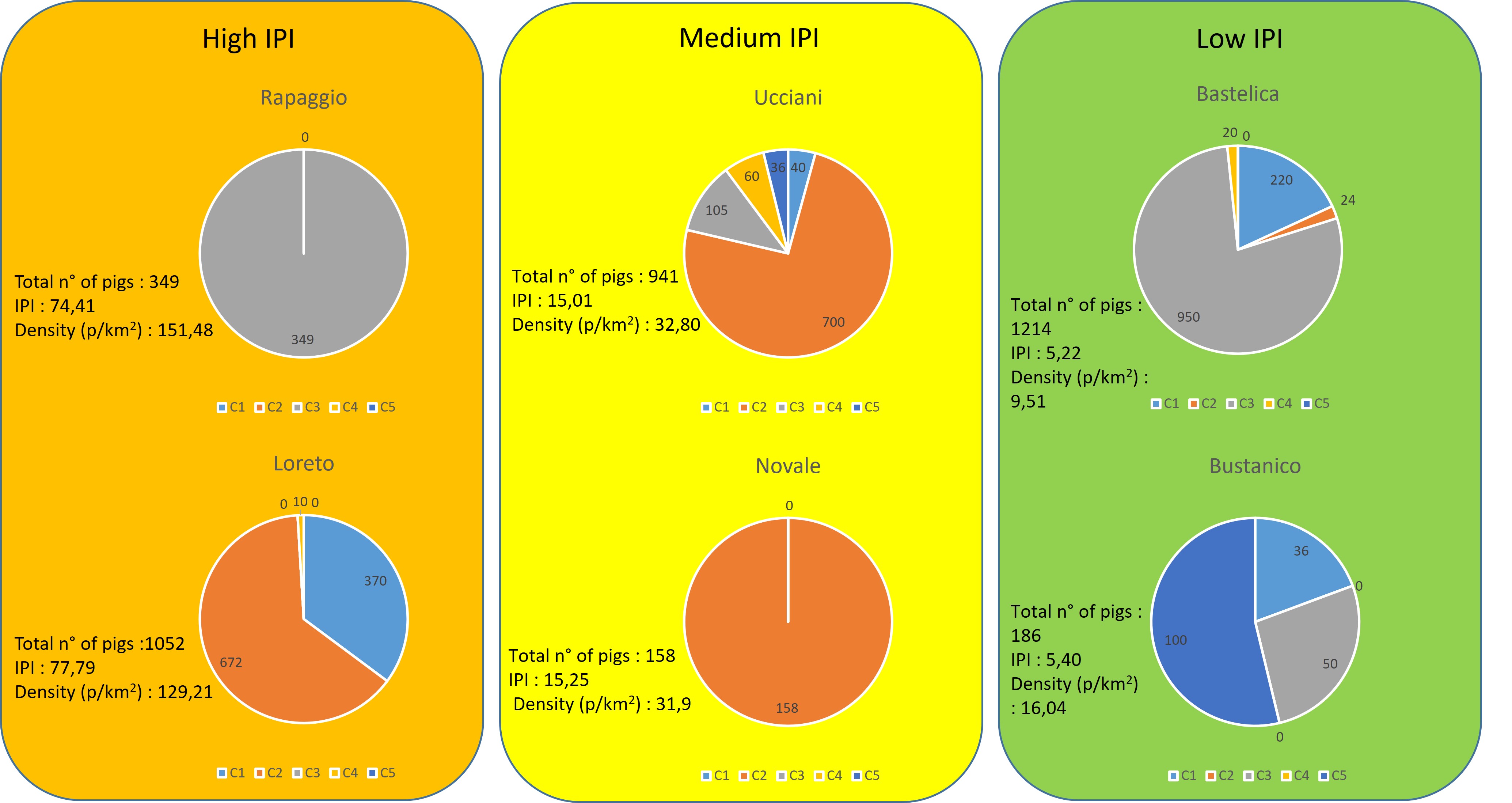

Supplement: Supplementary file 4 [file Image_3.JPEG]
